# Distinct Synaptic Vesicle Proteomic Signatures Associated with Pre- and Post-Natal Oxycodone-Exposure

**DOI:** 10.3390/cells11111740

**Published:** 2022-05-25

**Authors:** Katherine E. Odegaard, Gabriel Gallegos, Sneh Koul, Victoria L. Schaal, Neetha N. Vellichirammal, Chittibabu Guda, Andrea P. Dutoit, Steven J. Lisco, Sowmya V. Yelamanchili, Gurudutt Pendyala

**Affiliations:** 1 Department of Anesthesiology, University of Nebraska Medical Center, Omaha, NE 68198, USA; kodegaard@brookwoodschool.org (K.E.O.); gagallegos@unmc.edu (G.G.); skoul625@gmail.com (S.K.); vicki.schaal@unmc.edu (V.L.S.); andrea.dutoit@unmc.edu (A.P.D.); steven.lisco@unmc.edu (S.J.L.); 2 Department of Genetics, Cell Biology and Anatomy, University of Nebraska Medical Center, Omaha, NE 68198, USA; neethav@gmail.com (N.N.V.); babu.guda@unmc.edu (C.G.); 3 Child Health Research Institute, Omaha, NE 68198, USA

**Keywords:** synaptic vesicles, proteomics, in utero, post-natal, oxycodone, rodent model

## Abstract

The current opioid crisis, which has ravaged all segments of society, continues to pose a rising public health concern. Importantly, dependency on prescription opioids such as oxycodone (oxy) during and after pregnancy can significantly impact the overall brain development of the exposed offspring, especially at the synapse. A significant knowledge gap that remains is identifying distinct synaptic signatures associated with these exposed offspring. Accordingly, the overall goal of this current study was to identify distinct synaptic vesicle (SV) proteins as signatures for offspring exposed to oxy in utero (IUO) and postnatally (PNO). Using a preclinical animal model that imitates oxycodone exposure in utero (IUO) and postnatally (PNO), we used a quantitative mass spectrometry-based proteomics platform to examine changes in the synaptic vesicle proteome on post-natal day 14 (P14) IUO and PNO offspring. We identified MEGF8, associated with carpenter syndrome, to be downregulated in the IUO offspring while LAMTOR4, associated with the regulator complex involved in lysosomal signaling and trafficking, was found to be upregulated in the PNO groups, respectively. Their respective differential expression was further validated by Western blot. In summary, our current study shows exposure to oxy in utero and postnatally can impact the SV proteome in the exposed offspring and the identification of these distinct SV signatures could further pave the way to further elucidate their downstream mechanisms including developing them as potential therapeutic targets.

## 1. Introduction

In recent years, prevalent misuse of prescription opioids and a striking increase in the accessibility of illicit opioids have established what is defined as the opioid epidemic [1]. One of the most vulnerable groups is pregnant women since they are prescribed opioids such as morphine, buprenorphine, and methadone. These prescribed opioids have been demonstrated to cross the placenta as a result, possibly impacting the developing fetus [2,3,4]. However, limited data exist regarding the effects of in utero (IUO) or post-natal (PNO) exposure to oxycodone (oxy). Oxy is prescribed for many types of pain and can bind to mu- and kappa-opioid receptors [5]. Oxy also passes through the blood–brain barrier, allowing higher concentrations of oxy to accumulate in the brain, which can contribute to the analgesic properties as well as the risk for dependency and addiction [6,7,8].

Several studies have been conducted with rodent models to explore the harmful effects of gestational opioid use on the neurodevelopment of the offspring, but a knowledge gap exists regarding the impact of IUO or PNO oxy exposure on synaptogenesis [9]. Addictive drugs, such as opioids, produce significant and persistent changes in the synapse, which may explain their long-term effects [10]. While we have previously identified novel brain-derived extracellular vesicle miRNA signatures related to the neurodevelopment of PNO and IUO offspring [11], the present study aims to further investigate the effect of oxy exposure in inducing alterations in the synaptic vesicle (SV) proteome (chapter 2 of doctoral thesis; unpublished data) [12]. SVs (~40–50 nm in diameter), are found in the presynaptic terminal and act as a store for neurotransmitters [13]. Upon the arrival of an action potential, SVs docked and primed at the active zone of the presynaptic plasma membrane fuse with the membrane and release neurotransmitters into the synaptic cleft, after which the vesicles are retrieved via endocytosis, thus restoring the presynaptic vesicle pool [14]. With SVs contributing to about 5% of the total protein concentration of the mammalian central nervous system, characterizing their proteome composition could further help understand the dynamics of neurotransmission release which subsequently could impact brain function [13,14]. Several groups have used mass spectrometry and proteomics to compare protein expression profiles of parts of the synapse, such as synaptosomes, synaptic membranes, and SVs, to create a coherent map of the synapse proteome [15,16]. Such analyses of the synaptic proteome are critical in understanding the role of synaptic proteins in disease states such as drug addiction, as proteomic analysis allows for the global view of drug-induced changes within a specific proteome.

Using a pre- and post-natal oxy exposure rat model previously established in our lab, we employed a mass spectrometry-based proteomic approach to examine changes in the SV proteome from postnatal day 14 (P14) offspring from the in utero and post-natal oxy exposed offspring [11,17,18]. Subsequent pathway analysis of SV signatures highlighted several functional pathways affected by the differential expression of these SV signatures. The proteomics-based approach used in this study allows for in-depth research into the changes in SV signatures associated with perinatal oxy exposure. Further, the identification of associated functional pathways and disease states affected by the protein expression changes in these vesicles elucidates potential downstream effects that may continue to affect the development of IUO and PNO offspring.

## 2. Materials and Methods

### 2.1. Animals

Male and female Sprague Dawley rats were obtained from Charles River Laboratories Inc. (Wilmington, MA, USA). They were housed in a group in a 12-h light–dark cycle and fed *ad libitum*. The Institutional Animal Care and Use Committee of the University of Nebraska Medical Center approved all procedures and protocols (17-080). Procedures were performed in accordance with the National Institutes of Health Guide for the Care and Use of Laboratory Animals.

### 2.2. Oxycodone Treatment

The treatment paradigm previously established in our lab was followed [11,17]. Briefly, female (64–70 days of age) Sprague Dawley rats were treated with oxycodone HCl (Sigma Aldrich, St. Louis, MO, USA) dissolved in a saline vehicle via oral gavage. An ascending dosing procedure was used where doses of 10 mg/kg/day of oxy were orally-gavaged for 5 days followed by a 0.5 mg/kg/day escalation for 10 days until reaching a final dose of 15 mg/kg/day, after which females were mated with proven male breeders. The treatment regimen continued throughout mating, gestation, and parturition until weaning on post-natal day (P) 21. For the PNO paradigm, dams were orally-gavaged with 15 mg/kg/day of oxy only after parturition until weaning. The solo control group received no treatment in order to mimic a real-life scenario, i.e., normal drug-free mothers. For this study, offspring were sacrificed at P14, and brains were removed and stored at −80 °C.

### 2.3. Synaptic Vesicle Isolation

To investigate the effects of in utero and post-natal oxy exposure on synaptic transmission, we isolated synaptic vesicles (SVs) following the protocol designed by Ahmed, Holt, Riedel, and Jahn [13] with minor modifications. Briefly, one hemisphere of P14 rat brains (~500 mg) from each group (*n* = 6) was homogenized in 9 mL of ice-cold homogenization buffer (320 mM sucrose, 4 mM HEPES, freshly added protease inhibitor tablet) using a Wheaton Overhead Stirrer with ten strokes at 2k–3k RPM, and 100 µL of homogenate was saved for use in Western blot. The homogenized mixture was centrifuged at 2700 RPM (1000× *g*) for 11 min at 4 °C, and the pellet (P1; cell fragments and nuclei) was discarded. The supernatant was centrifuged at 11,000 RPM (15,000× *g*) for 16 min at 4 °C. The resulting supernatant (S2) was collected in a 15 mL tube. The pellet (P2; synaptosomes) was washed carefully in 1 mL of homogenization buffer, leaving the brown center of the pellet (mitochondria) remaining on the tube, and100 µL of P2 was saved for use in Western blot. The resuspended P2 was transferred to a tight-fitting glass-Teflon homogenizer, and 9 mL of ice-cold double-distilled water was added to perform osmotic lysis. Osmotic lysis was accomplished by performing three strokes at 2500 RPM. Then 50 µL of 1 M HEPES and protease inhibitors were added immediately after the last stroke. The osmotic lysis product was centrifuged at 12,000 RPM (17,000× *g*) for 19 min at 4 °C in a swinging Ti41 ultracentrifuge rotor (Beckman Coulter, Brea, CA, USA). The resulting supernatant (LS1) was combined with S2. The LS1/S2 mixture was centrifuged at 20,000 RPM (48,000× *g*) for 29 min at 4 °C in a swinging Ti41 ultracentrifuge rotor. The resulting supernatant (CS1) was transferred to a tight-fitting glass-Teflon homogenizer and homogenized using 3–5 strokes at 2k–3k RPM. To ensure disruption of SV clusters, the homogenized CS1 was drawn through a 20-gauge hypodermic needle attached to a 10 mL syringe and expelled through a 27-gauge needle. Then 5 mL of CS1 was carefully layered onto a 5.5 mL 0.7 M sucrose cushion and centrifuged at 38,000 RPM (133,000× *g*) for 1 h at 4 °C in a swinging Ti41 ultracentrifuge rotor. 

After centrifugation, 350 µL fractions were collected, starting from the top of the gradient, resulting in thirty 350 µL fractions. The white pellet at the bottom of the gradient was resuspended in 200 µL of homogenization buffer, and 50 µL of each fraction and the resuspended pellet were collected for use in a dot blot. The remaining resuspended pellet was homogenized using a handheld homogenizer. Fractions 16–30 and the pellet were combined and centrifuged at 245,000× *g* for 2 h and 35 min at 4 °C in a swinging Ti41 ultracentrifuge rotor. These fractions were used based on purity verification using dot blotting. The supernatant was discarded, and the pellet was resuspended in 200 µL of 1X PBS. The ultracentrifugation tube was rewashed with 100 µL of 1X PBS to ensure complete reconstitution of the pellet. The resuspended SVs were drawn through a 27-gauge hypodermic needle attached to a 1 mL syringe and expelled, and 100 µL of the SV was saved for Western blotting. SVs were stored at −80 °C. Protein quantification of the isolated SVs was carried out using Pierce BCA protein assay (Thermo Fisher Scientific, Waltham, MA, USA).

### 2.4. Dot Blot

To ensure the purity of the isolated SVs, 2 µL of each of the 30 separated fractions and the pellet from the SV isolation were blotted onto a nitrocellulose membrane divided into grids. Samples were added slowly, so the area of the absorbed drop of the sample was 2–4 mm in diameter. The membrane was allowed to dry before blocking non-specific sites using 5% BSA in TBS-T for 30 min to 1 h. The membrane was incubated with primary antibody diluted in 0.1% BSA in TBS-T for 30 min at room temperature. Anti-PSMC6 (1:1000; Abcam, Cambridge, UK), a proteasome marker, was used to determine fraction purity and ensure no fractions with proteasome contaminants continued into the sucrose cushion step of the isolation protocol. After incubation, the membranes were washed three times with TBS-T for 5 min each. The secondary antibody was diluted according to the manufacturer’s protocol in 0.1% BSA in TBS-T and added to the membrane for 30 min at room temperature. Secondary antibodies were HRP-conjugated anti-rabbit IgG (Thermo Scientific). The membrane was washed three times with TBS-T for 5 min each. Membranes were allowed to sit in SuperSignal West Pico Chemiluminescent Substrate (Thermo Scientific) for 1 min before imaging with the Azure cSeries Imager (Azure Biosystems, Dublin, CA, USA).

### 2.5. Western Blot

SVs (10–20 µg) from each animal were loaded into 4–12% Bis-Tris wells (Invitrogen, Waltham, MA, USA) under reducing conditions, followed by transfer to a nitrocellulose membrane using iBlot2 (Invitrogen) and immunodetection. Nonfat milk (5%) was used to block nonspecific antibody binding (Thermo Fisher Scientific, Waltham, MA, USA). After blocking, membranes were incubated overnight at 4 °C with a primary antibody. Primary antibodies included GAPDH (Invitrogen), PSD95 (Invitrogen), GFAP (Sigma-Aldrich, St. Louis, MO, USA), SYP (ThermoFisher), VGLUT1 (Santa Cruz Biotech, Dallas, TX, USA), and SNAP25 (Synaptic Systems, Goettingen, Germany). MEGF8 (Bioworld Technology, St. Louis Park, MN, USA) and LAMTOR4 (Cell Signaling, Danvers, MA, USA) were additionally selected from the proteomic analysis for post-validation. Secondary antibodies were HRP-conjugated anti-rabbit IgG (Thermo Scientific) and HRP-conjugated anti-mouse IgG (Thermo Scientific). Primary and secondary antibody dilutions were done according to the manufacturer’s suggestion and are shown in Supplementary Table S1. Blots were developed using Azure cSeries Imager (Azure Biosystems, Dublin, CA, USA) with SuperSignal West Pico Chemiluminescent Substrate (Thermo Scientific). 

### 2.6. Mass Spectrometry

Briefly, 50 µg of protein per SV sample from six biological replicates per group was taken, and detergent was removed by chloroform/methanol extraction. With 100 mM ammonium bicarbonate, the protein pellet was resuspended followed by digestion with MS-grade trypsin (Thermo Fisher) 37 °C overnight. PepClean C18 spin columns (Thermo Scientific) were used to clean peptides. Cleaned peptides were re-suspended in 2% acetonitrile (ACN) and 0.1% formic acid (FA), and 500 ng of each sample was loaded onto trap column Acclaim PepMap100, 75 µm × 2 cm C18 LC Columns (Thermo Scientific) at a flow rate of 4 µL/min. Samples were then separated with a Thermo RSLC Ultimate 3000 (Thermo Scientific) on a Thermo Easy-Spray PepMap RSLC C18, 75 µm × 50 cm C-18 2 µm column (Thermo Scientific) with a step gradient of 4–25% solvent B (0.1% FA in 80% ACN) from 10–130 min and 25–45% solvent B for 130–145 min at 300 nL/min and 50 °C with a 180-min total run time. Following separation, the Thermo Orbitrap Fusion Lumos Tribrid (Thermo Scientific) mass spectrometer, in a data-dependent acquisition mode, was used to analyze the eluted peptides. An MS scan (from *m/z* 350–1800) was acquired in the Orbitrap with a resolution of 120,000. The AGC target for MS1 was set as 4 × 10^5^ and ion filling time set as 100 ms. In order to isolate the most intense ions with a charge state of 2–6, a 3 s cycle was performed followed by fragmented using HCD fragmentation with 40% normalized collision energy and detected at a mass resolution of 30,000 at 200 *m/z*. The AGC target for MS/MS was set as 5 × 10^4^ and ion filling time set at 60 ms; dynamic exclusion was set for 30 s with a 10-ppm mass window. In order to identify proteins a search was conducted where the MS/MS data were viewed against the Swiss–Prot *Rattus norvegicus* protein database downloaded in May 2019, using the in-house mascot 2.6.2 (Matrix Science, Canton, MA, USA) search engine. The search criteria consisted of full tryptic peptides with a maximum of two missed cleavage sites. The variable modifications included acetylation of protein N-terminus and oxidized methionine. The fixed modification was carbamidomethylation of cysteine. Additional parameters set include the precursor mass tolerance threshold, which was set at 10 ppm, and the maximum fragment mass error which was set at 0.02 Da. A false discovery rate (FDR) of ≤1% was used to determine the significant threshold of the ion score. Progenesis QI proteomics 4.1 (Nonlinear Dynamics, Milford, MA, USA) was then utilized to perform qualitative analysis.

### 2.7. Bioinformatic Analysis

Proteins were identified as differentially expressed if the FDR-corrected *p*-value was ≤0.05 and displayed a fold change ≥ 2. Heatmaps of the top 25 proteins in each comparison were plotted using the function heatmap.2 in the R (version 3.6.0) package gplots. Cytoscape plugin ClueGO was used to perform gene ontology (GO) analysis of differentially expressed proteins [19]. Biological processes, molecular functions, and KEGG pathways were included for GO enrichment analysis. Enriched disease-associated pathways were identified using the Ingenuity Pathway Analysis (IPA) software (Ingenuity^®^ Systems, Redwood City, CA, USA). Canonical pathway analysis in IPA was performed by comparing the differentially expressed proteins against known canonical pathways (signaling and metabolic) within the IPA database.

### 2.8. Statistical Analyses

A student *t*-test was performed to identify proteins with significant differences between groups (saline vs. PNO and saline vs. IUO). Proteins were considered significant if they had at least two unique peptides and a *p*-value < 0.05. Significant protein fold change differences were determined through an unpaired *t*-test. All statistical tests were performed with GraphPad Prism (La Jolla, CA, USA); data are represented as the mean ± SEM on the graphs.

## 3. Results

### 3.1. Purity and Validation of Synaptic Vesicle Samples

**Figure 1:**
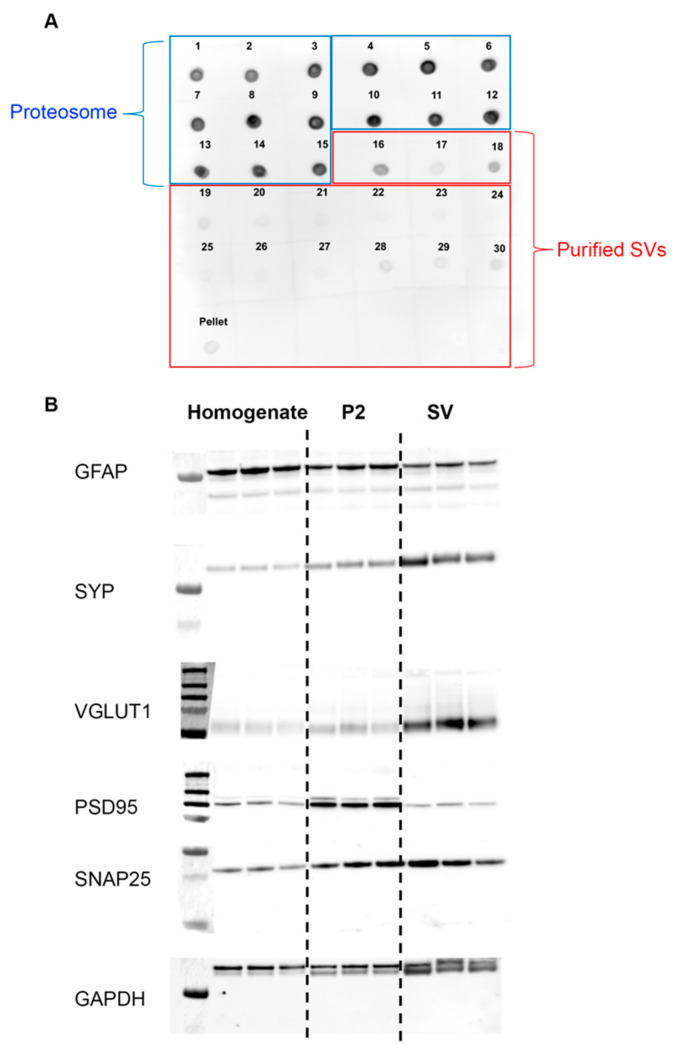
SVs purity validation. (**A**) Dot blot analysis of SVs from P14 saline rats shows purified SVs within the 16–30 fraction range. (**B**) Western blot analysis of SVs from P14 saline rats shows the expression of positive SV markers (VGLUT1, SYP, SNAP25) and negative markers (GFAP, PSD95).

To verify our isolate contained only purified SVs, we performed dot blot analyses on isolated fractions collected during different stages of our protocol. To ensure no proteasome contaminants remained in our sample, we conducted a dot blot analysis using the proteasome marker anti-PSMC. Of the 30 fractions and the pellet collected from our protocol, we found the distribution of proteasome contaminants was higher in fractions 1–15 compared to fractions 16–30 and the pellet (Figure 1A). Thus, only fractions 16–30 and the pellet were used for our SV isolation protocol.

Further, we validated the purity of our isolated SVs through Western blot analysis. We compared the expression levels of known synaptic proteins present in the homogenate, synaptosome (P2), and SV fractions (Figure 1B). Known synaptic proteins, such as VGLUT1, SYP, and SNAP25, present on SVs, were enriched in the final SV fractions. Proteins not specific to the SV, such as GFAP and PSD95, were enriched in the homogenate and P2 fractions. After validating the purity of our SV product, samples from each group were sent for mass spectrometry. 

### 3.2. Differential Expression of Synaptic Vesicle Proteins

**Figure 2:**
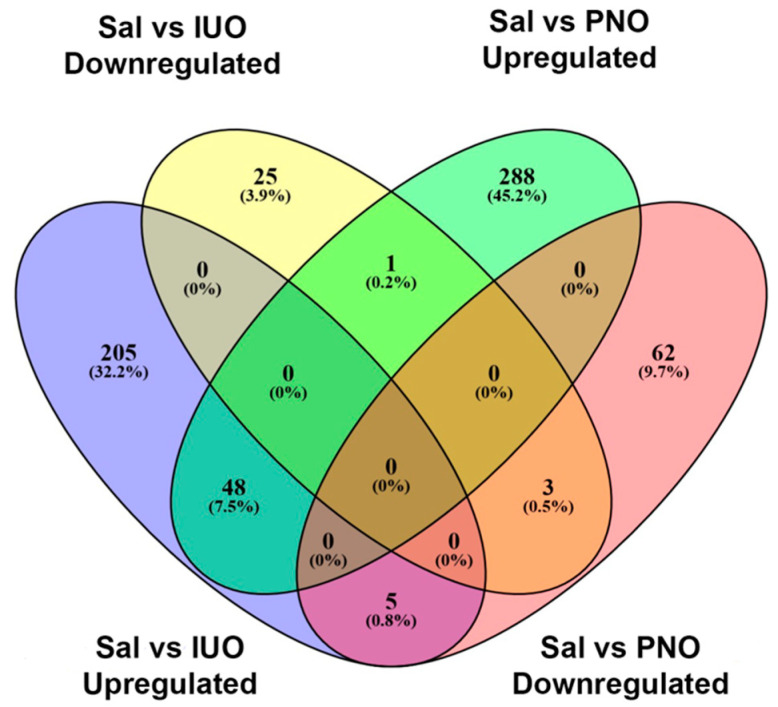
Venn diagram showing the differentially expressed SV proteins determined from the proteomics screen.

To examine changes in the SV proteome resulting from in utero and post-natal oxy exposure, purified SVs were sent for proteomic analysis using quantitative mass spectrometry. A total of 4135 and 3804 proteins were identified between IUO and PNO, respectively. Further employing a criterion of 2+ unique peptides and *p* < 0.05, 865 and 810 proteins were found to be differentially expressed in both the IUO and PNO groups when compared to saline controls (Supplementary Tables S1 and S2). Figure 2 shows a Venn diagram of the differentially expressed proteins that were uniquely up- or downregulated in the IUO and PNO groups. Of the differentially expressed proteins in the PNO group compared to controls, 288 were upregulated, and 62 were downregulated. In the IUO group, 205 proteins were upregulated while 25 proteins were downregulated. Further, of the upregulated proteins in both PNO and IUO comparisons with controls, 48 proteins were common. Of the downregulated proteins, 3 proteins were common: RTCA, LYNX1, and JAGN1. Furthermore, when comparing the upregulated proteins of the IUO with the downregulated proteins of the PNO, 5 proteins were common: SLITRK1, GSTK1, PRPF6, APOO, and CTTN. When comparing the upregulated proteins of the PNO with the downregulated proteins of the IUO, only one protein was common which was ERH. Comparisons between the IUO and PNO groups revealed 17 proteins to be upregulated and 6 proteins to be downregulated in the IUO animals (Supplemental Table S3). 

**Figure 3:**
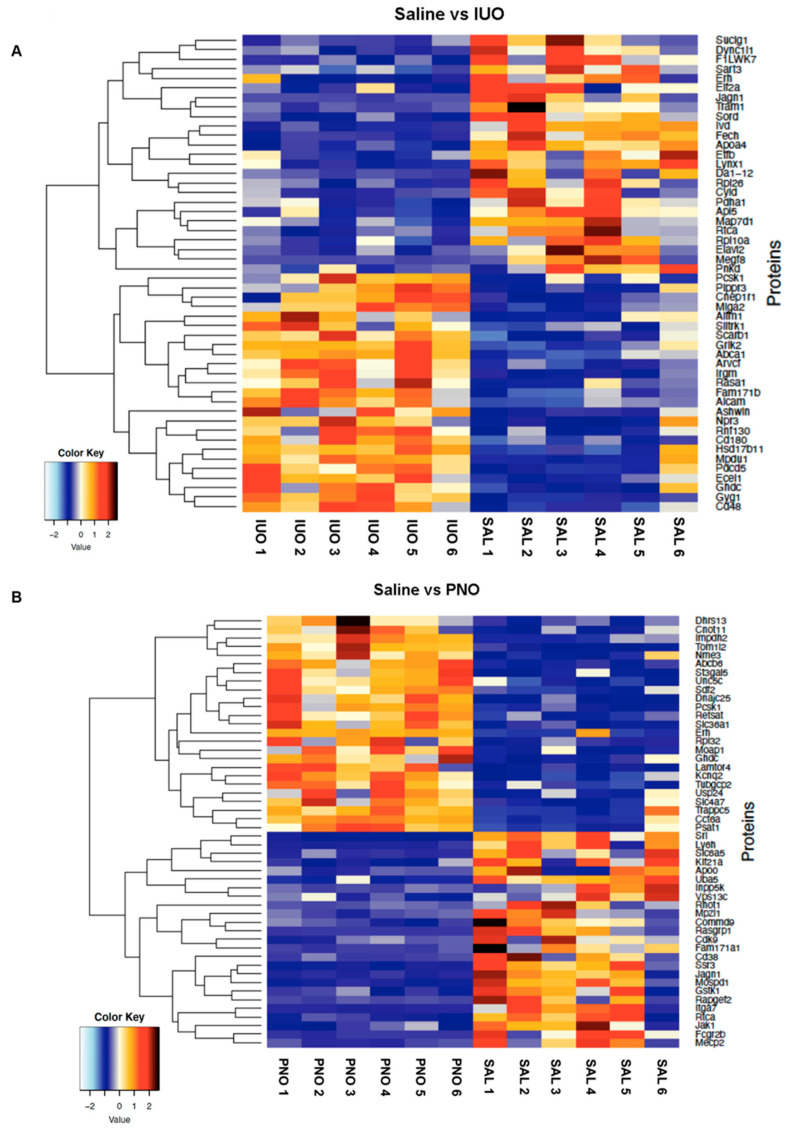
Heatmap showing the top differentially expressed SV proteins between IUO (**A**) and PNO (**B**) groups compared to the saline.

A heatmap was then created based on the significant differentially expressed SV proteins between IUO and PNO groups versus the saline group as shown in Figure 3A,B.

**Figure 4:**
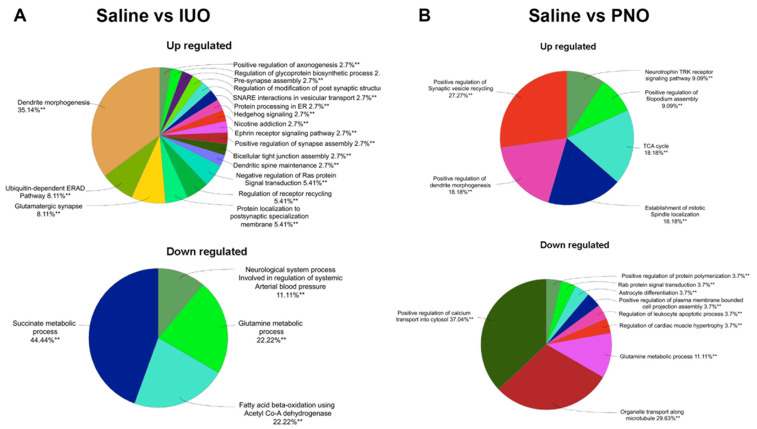
ClueGO analysis shows the different biological processes in the IUO (**A**) and PNO (**B**) groups. Group term *p*-value is represented by asterisks for each category. ** *p* < 0.01.

A ClueGo analysis of the differentially expressed proteins highlighted several pathways that were affected in the PNO and IUO groups. In the IUO group, dendrite morphogenesis, glutamatergic synapse, pre-synapse assembly, and protein localization to postsynaptic specialization membrane were among the biological processes affected by the upregulation of SV proteins (Figure 4A). The biological processes that were affected by the downregulation of SV proteins in the IUO group includes succinate metabolic process, neurological system process involved in the regulation of systemic arterial blood pressure, glutamine metabolic process, and fatty acid beta-oxidation using acetyl Co-A dehydrogenase. In the PNO group, positive regulation of synaptic vesicle recycling and dendrite morphogenesis, the establishment of mitotic spindle localization, TCA cycle, and neurotrophin TRK receptor signaling pathway were among the biological processes affected by the upregulation of SV proteins (Figure 4B). The biological processes affected by the downregulation of SV proteins in the PNO group include the positive regulation of calcium transport into the cytosol, organelle transport along the microtubule, glutamine metabolic process, and Rab protein signal transduction. 

**Figure 5:**
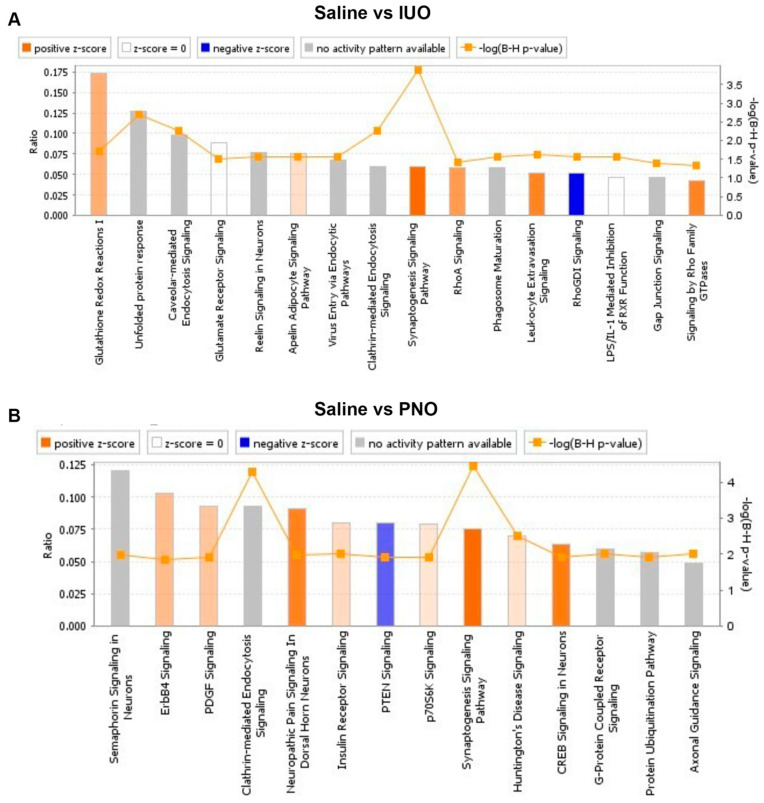
Ingenuity pathway analysis (IPA). IPA reveals several enriched canonical disease-associated pathways within both PNO (**A**) and IUO (**B**) comparisons. The pathways are ranked by the negative log of the FDR corrected *p*-value of the enrichment score and color coded according to the Z-score. A significantly increased pathway activity is indicated by a positive Z-score represented by the orange bars and an overall decrease in pathway activity is represented by a negative Z-score represented by blue bars. Gray bar represents enriched pathways with no predicted activity change.

Further, enriched disease-associated pathways were identified using the Ingenuity Pathway Analysis (IPA) software (Figure 5A,B). Compared to the saline control, the top canonical pathways affected in the IUO group included the synaptogenesis pathway, clathrin-mediated endocytosis signaling, caveolar-mediated endocytosis signaling, and glutathione redox reactions. Pathways associated with neurological disease, inflammatory response, and respiratory disease were among the disease-associated pathways affected by IUO treatment. Physiological and molecular functions, such as nervous system development, behavior, and cell–cell signaling, were also affected in the IUO group. Further, pathways associated with cardiac, liver, and renal abilities were affected with IUO treatment. In the PNO group, we identified canonical pathways associated with the synaptogenesis pathway, clathrin-mediated endocytosis signaling, Huntington’s disease signaling, insulin receptor signaling, and axonal guidance signaling. Additionally, pathways associated with neurological disease, hereditary disorders, psychological disorders, and skeletal and muscular disorders were also affected in the PNO group. Physiological and molecular pathways, such as those involved in nervous system development and function, behavior, cellular development, and cellular growth and proliferation, were also affected in these animals. Several pathways associated with heart failure, liver dysfunction, and renal issues were also impacted by PNO treatment. Overall, both IUO and PNO animal SV proteomes denoted several differentially regulated proteins that impact biological pathways, particularly those associated with several disease states. Similarly, ClueGO analysis comparing the IUO and PNO groups identified actin filament binding, post synapse organization, and maintenance of bipolar cell polarity to be significantly affected in the IUO animals (Supplemental Figure S1). 

**Figure 6:**
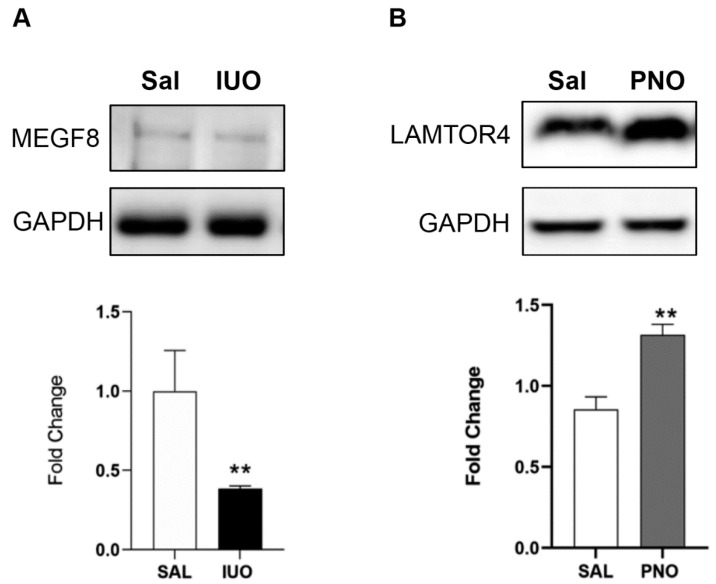
Validation of regulation of MEGF8 and LAMTOR4. Representative Western blot analysis on SV markers isolated from PNO, IUO, and saline shows the downregulation of MEGF8 in the IUO group (**A**) and upregulation of LAMTOR4 in the PNO group (**B**). Data represented as mean ± SEM, *n* = 5–6 animals per group, ** *p* < 0.01 as determined by an unpaired *t*-test after Welch’s correction.

Further, the heatmap revealed analysis-ready hits that were significantly up- or down-regulated in the groups. Of these, MEGF8, associated with carpenter’s syndrome, was downregulated in the IUO SVs, and LAMTOR4, a regulator of microglial lysosomes, was upregulated in the PNO SVs [20,21,22]. Our Western blots validated these results, showing a lower expression of MEGF8 in IUO SVs and a higher expression of LAMTOR4 in PNO SVs compared to the controls (Figure 6A,B).

## 4. Discussion

Drugs of abuse, such as amphetamine, cocaine, nicotine, and morphine, have been shown to alter the structure of dendrites and dendritic spines on cells in regions of the brain associated with reward, judgment, and inhibitory control. Robinson et al. suggested that this structural plasticity associated with exposure to drugs of abuse reflects a reorganization of synaptic connectivity patterns that alters their operation, thus contributing to the detrimental effects of drug use, namely addiction [10]. Alterations in the synaptic abilities in one area of the brain may strengthen or weaken connections with other brain regions, possibly driving distinct aspects of addictive behavior [23]. As opposed to drugs of abuse, opioids such as morphine and oxycodone are commonly administered in clinical settings to patients of all ages including during pregnancy and the neonatal period. Chronic morphine studies have shown alterations in gene expression, synapse morphology, synaptic transmission, and synaptic protein profiles, but studies regarding oxy are still lacking [24]. With regard to synaptic plasticity, our lab has previously shown that the number of dendritic spines decreased with PNO and IUO exposure [11]. In the same study, we identified novel extracellular vesicle signatures associated with PNO or IUO treatment that may impact synaptogenesis in these offspring. The present study sought to further this investigation of the impact of PNO and IUO exposure on synaptic development through the use of synaptic vesicles, identifying novel SV signatures associated with PNO and IUO exposure. We also found that the differentially expressed proteins associated with the SVs impact functional pathways and disease states. As mentioned in our previous work, the PNO and IUO groups are clinically relevant, thus lending translational significance to our study [11].

From the purified SVs isolated from the P14 PNO and IUO rat brains, several functional pathways and disease states were suggested to be affected by the differentially regulated protein contents of the SVs. Of the several pathways impacted by PNO and IUO exposure, axon guidance and axon fasciculation were particularly interesting. Human studies have shown that prenatal opioid exposure can result in abnormal tract development in newborns that persists at 12 to 15 years of age [25,26]. Additionally, infants born with prenatal opioid exposure had alterations or abnormalities in the white matter, such as punctate white matter lesions [27]. In preclinical studies, chronic oxy exposure altered the white matter of rats through axonal track deformation, a decrease in the size of axonal fascicles, loss of myelin basic protein, and buildup of amyloid precursor protein [28]. Proteomic investigations of morphine-regulated changes in the synapse have shown signaling, vesicle trafficking, cytoskeletal proteins, energy metabolism, signal transduction, synaptic transmission, and cell adhesion all to be affected [24,29,30,31,32,33]. Similar pathways were shown to be affected in both PNO and IUO groups of our study, specifically, pathways associated with vesicle docking, membrane adhesion, signaling, synapse assembly, and vesicular transport.

Increasing evidence suggests that the administration of opioids is associated with many medical diseases including those related to the pathophysiology of neurological disorders [34]. Opioid use has been connected to mental health disorders, and specifically, a large proportion of prenatally exposed adolescents have been shown to have experiences with major depressive episodes, alcohol abuse, and attention deficit hyperactivity disorder [35]. In our PNO group, Huntington’s disease signaling was affected by oxy exposure. Interestingly, in patients with Huntington’s disease, individuals who abused substances had a significantly earlier age of motor onset [36]. In this study, both PNO and IUO exposures were also associated with a number of alterations in molecular pathways that lead to renal complications. Several human studies showcasing opioid overdose have demonstrated that opioid use has been associated with acute kidney injury [37]. Additionally, chronic morphine use was associated with an increased risk of both renal and hepatic damage [38]. Liver disease associated with PNO and IUO groups may stem from the metabolism of oxy in the liver. Verna et al. suggest that the metabolic pathways involved in opioid metabolism may contribute to or worsen liver injury [39]. δ-opioid receptors, which contribute significantly to cellular development and are abundant in liver tissue, have been shown to affect the initiation and progression of liver disease. Additionally, histopathologic examination of hepatocytes from rat models of chronic opioid use exhibited sinusoidal dilatation, perivenular ballooning degeneration extending to the midzonal region, perivenular necrosis, hemorrhage, and focal microvesicular steatosis [38]. With regard to cardiac diseases, which were associated with both PNO and IUO groups, a recent study found that coronary artery disease was significantly higher in patients with opioid use than in controls, suggesting opioid use may be an important risk factor in this disease [40]. Further, opiate consumption has been linked to hypertension and diabetes mellitus, and evidence suggests that chronic opioid use may increase the risk of cardiovascular diseases [41]. Taken together, these results highlight the importance of understanding the long-term effects of perinatal exposure to opioids, as a number of complications and disease states have been associated with the administration of opioids.

The downregulation of MEGF8 and upregulation of LAMTOR4 in the IUO and PNO SVs, respectively, revealed in our differential expression analyses were particularly interesting. MEGF8 is a single-pass type I membrane protein that contains several EGF-like domains [21]. Defects in the gene that encodes MEGF8 result in carpenter syndrome, which is defined as congenital malformation described by craniosynostosis and polysyndactyly [20]. Interestingly, loss of MEGF8 disrupts axon guidance in the peripheral nervous system, leading to limb, heart, and left-right patterning defects [42]. As mentioned before, human newborns with prenatal opioid exposure had alterations in the white matter, and preclinical models of chronic oxy administration have revealed deformation of axonal tracks, reduction of the size of axonal fascicles, loss of myelin basic protein, and accumulation of amyloid precursor protein [27,28]. Perhaps the downregulation of MEGF8 may contribute to these deficits in axonal development reported with prenatal opioid exposure.

LAMTOR4 is part of the Rag complex, a scaffold protein complex comprised of LAMTOR subunits 1–5 that senses amino acids and lips and integrates growth factor signaling [43]. This “Ragulator” complex also controls the activity of the mTOR complex 1 (mTORC1) on the lysosome [44]. Interestingly, mTORC1 is required to initiate and maintain chronic pain and opioid-induced tolerance/hyperalgesia [45]. Additionally, it has been suggested that prenatal opioid exposure is the next neonatal inflammatory disease [46]. Intriguingly, LAMTOR4 is essential for microglial development; zebrafish lacking LAMTOR4 were shown to have a reduction in microglia [22]. Further investigation into LAMTOR4 and the Ragulator complex in the context of PNO exposure is needed to understand its role in opioid exposure.

## 5. Conclusions

Overall, our study found that IUO and PNO oxy exposure alter the proteome of SVs, impacting the synaptic abilities of these offspring. These results suggest that in utero and post-natal exposure to oxy can alter the proteome of SVs, thus influencing signaling pathways and potentially increasing the vulnerability of these offspring to disease in the future.

## Data Availability

All the data is contained within the article and the Supplementary Materials.

## Supplementary Materials

The following supporting information can be downloaded at: https://www.mdpi.com/article/10.3390/cells11111740/s1, Figure S1: (A) Heatmap showing the top differentially expressed SV proteins between IUO and PNO groups. (B) ClueGO analysis comparing the IUO and PNO groups shows key biological processes associated with actin filament binding, post synapse organization, and maintenance of bipolar cell polarity to be significantly affected in the IUO animals. (C) Ingenuity pathway analysis showing the canonical disease-associated pathways between the IUO and PNO groups; Figure S2: Individual western blots on isolated synaptic vesicles from all animals used in the study. Boxed blots are shown in the manuscript. S-Saline, N-Post natal oxy (PNO), IUO-in utero oxy; Table S1: IUO vs. Sal; Table S2: PNO vs. Sal; Table S3: IUO vs. PNO.
