# Correction: Odegaard et al. Distinct Synaptic Vesicle Proteomic Signatures Associated with Pre- and Post-Natal Oxycodone-Exposure. *Cells* 2022, *11*, 1740

**DOI:** 10.3390/cells13161375

**Published:** 2024-08-19

**Authors:** Katherine E. Odegaard, Gabriel Gallegos, Sneh Koul, Victoria L. Schaal, Neetha N. Vellichirammal, Chittibabu Guda, Andrea P. Dutoit, Steven J. Lisco, Sowmya V. Yelamanchili, Gurudutt Pendyala

**Affiliations:** 1Department of Anesthesiology, University of Nebraska Medical Center, Omaha, NE 68198, USA; kodegaard@brookwoodschool.org (K.E.O.); gagallegos@unmc.edu (G.G.); skoul625@gmail.com (S.K.); vicki.schaal@unmc.edu (V.L.S.); andrea.dutoit@unmc.edu (A.P.D.); steven.lisco@unmc.edu (S.J.L.); 2Department of Genetics, Cell Biology and Anatomy, University of Nebraska Medical Center, Omaha, NE 68198, USA; neethav@gmail.com (N.N.V.); babu.guda@unmc.edu (C.G.); 3Child Health Research Institute, Omaha, NE 68198, USA

## Data Availability Statement

In the original publication [1], there was an error in the Data Availability Statement. Initially, the statement declared ‘Not applicable’. The corrected Data Availability Statement is below.

“All the data is contained within the article and the Supplementary Materials.”

## References

The authors would like to remove the reference number 40, “Tang, B.; Zhang, Y.; Liang, R.; Yuan, P.; Du, J.; Wang, H.; Wang, L. Activation of the δ-opioid receptor inhibits serum deprivation-induced apoptosis of human liver cells via the activation of PKC and the mitochondrial pathway. *Int. J. Mol. Med.*
**2011**, *28*, 1077–1085”, due to the retraction of that article. With this correction, the order of some references has been adjusted accordingly.

The authors state that with these corrections, the scientific conclusions are unaffected. These corrections were approved by the Academic Editor. The original publication has also been updated.
