# Supplementary material for: Distinct Synaptic Vesicle Proteomic Signatures Associated with Pre- and Post-Natal Oxycodone-Exposure
Source: Cells. 2022 May 25;11(11):1740. doi: 10.3390/cells11111740 (PMC9179517; doi:10.3390/cells11111740)
Supplement: Supplementary file 1 [file cells-11-01740-s001.zip › cells-1665631-supplementary/Supplement Figures S1&S2.pdf]

Supplemental Information.

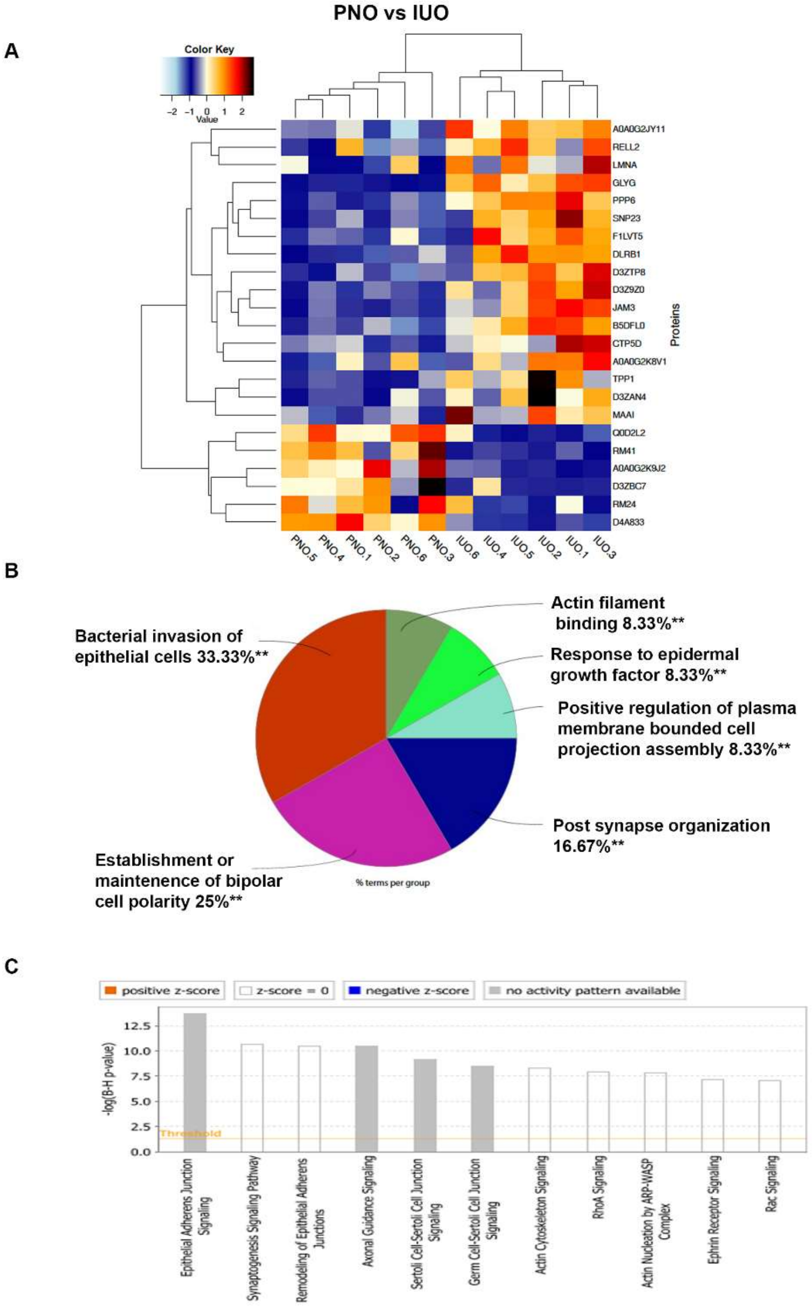

Supplementary Figure S1: (A) Heatmap showing the top differentially expressed SV proteins between IUO and PNO groups. (**B**) ClueGO analysis comparing the IUO and PNO groups shows key biological processes associated with actin filament binding, post synapse organization, and maintenance of bipolar cell polarity to be significantly affected in the IUO animals. **(C**) Ingenuity pathway analysis showing the canonical disease-associated pathways between the IUO and PNO groups.

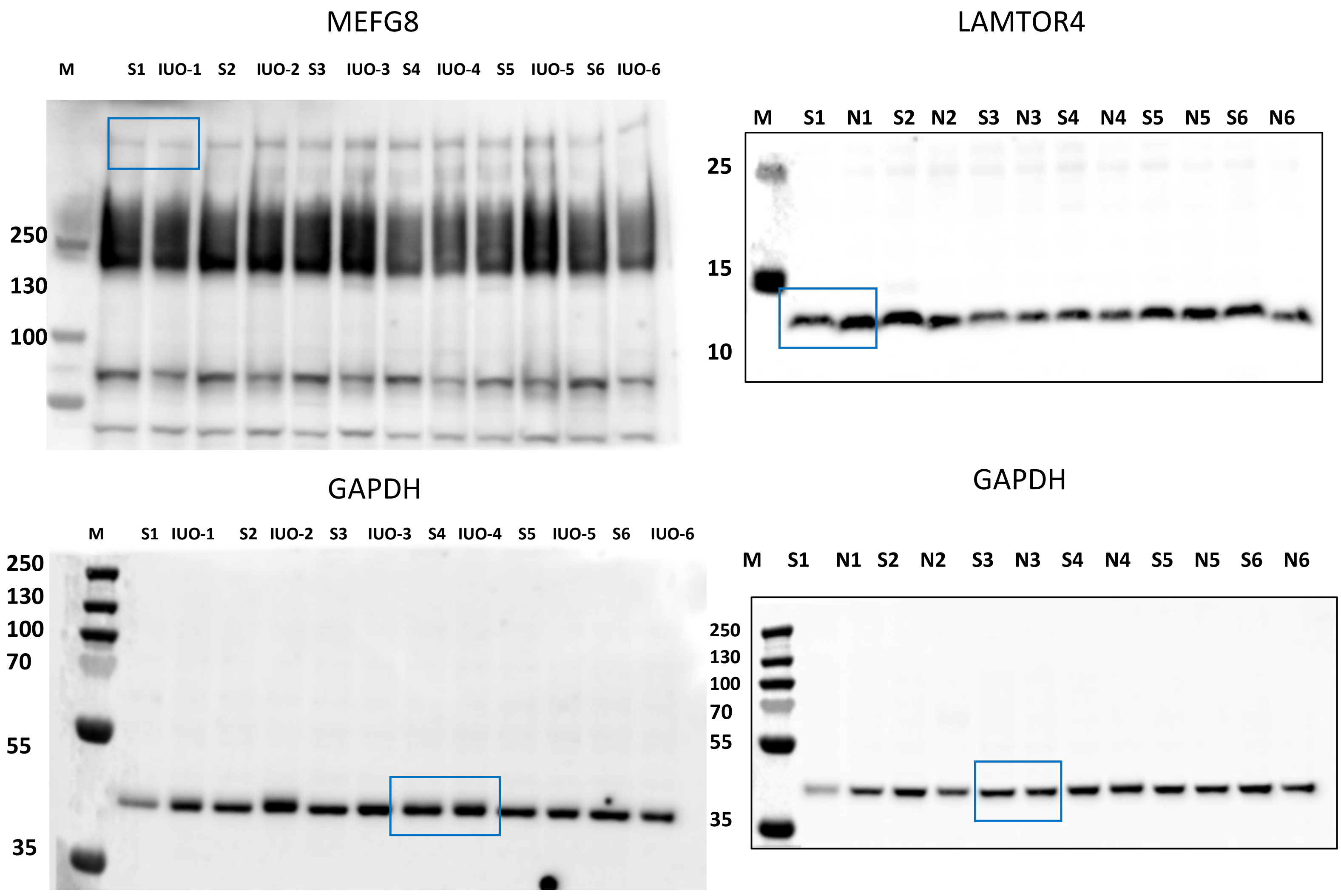

Supplementary Figure S2: Individual western blots on isolated synaptic vesicles from all animals used in the study. Boxed blots are shown in the manuscript. S-Saline, N-Post natal oxy (PNO), IUO-*in utero* oxy.
